# Genomic analysis of a ginger pathogen *Bacillus pumilus* providing the understanding to the pathogenesis and the novel control strategy

**DOI:** 10.1038/srep10259

**Published:** 2015-05-19

**Authors:** Yihui Yuan, Meiying Gao

**Affiliations:** 1 Key Laboratory of Agricultural and Environmental Microbiology, Wuhan Institute of Virology, Chinese Academy of Sciences, Wuhan, P.R. China

## Abstract

*Bacillus pumilus* has been widely identified as a pathogen of plant and human, while the genetic information is rarely available for pathogenic *B. pumilus* strains. *B. pumilus* GR8 is a pathogen that causes ginger rhizome rot disease by invading ginger rhizome parenchymatous tissues, growing in the extracellular space, and producing plant cell wall-degrading enzymes to destroy ginger cells. In this study, the genome of GR8 was sequenced and characterized. This genome was the third completely sequenced genome of the *B. pumilus* species, and it exhibited high similarity to the genome of the *B. pumilus* strain B6033. The genome of GR8 was 3.67 Mb in length and encoded 3,713 putative ORFs. Among these predicted proteins, numerous plant cell wall-degrading enzymes and several proteins associated with invading and adapting to the environment in the extracellular space of the ginger rhizome parenchymatous tissue were found. The GR8 genome contained only one restriction-modification system and no CRISPR/Cas system. The lack of phage-resistant system suggested that phages might be potential agents for the control of GR8. The genomic analysis of GR8 provided the understanding to the pathogenesis and the phage-control strategy of pathogenic *B. pumilus* strains.

*Bacillus pumilus* is a bacterial species belonging to the *Bacillus subtili*s group^1^. Based on its abilities in producing vanillin, keratinase, xylanase, alkaline serine protease and several other bioactive substances^2^,^3^,^4^,^5^, *B. pumilu*s is widely used in industrial processes, such as the production of several traditional fermented foods, the treatment of wastewater and the degradation of environmental pollutants^6^,^7^. More recently, *B. pumilus* found in healthy plant tissue was reported with properties in promoting plant growth by enhancing the uptake of nutrients, nitrogen fixation, interaction with symbiotic microorganisms and producing antimicrobial agents against pathogenic bacteria and fungi as well as by reducing metal toxicity^8^,^9^,^10^,^11^. According to previous reports, several other *B. pumilus* strains are also used as probiotics for animals^12^. However, numerous *B. pumilus* strains have been reported to cause plant and human diseases. Strains of *B. pumilus* associated with foodborne illness and from clinical environments have been isolated, and several *B. pumilus* strains, such as strain F4791/87 and F4552/87, have been found to produce toxicin that are harmful to humans^13^,^14^. Two *B. pumilus* isolates have been identified that cause severe sepsis of neonatal infants^15^. Six *B. pumilus* strains isolated from Egypt have been reported to cause mango tree disease, and one *B. pumilus* strain isolated from China is associated with leaf and twig dieback of Asian pear trees^16^,^17^.

In previous studies, the genomes of ten strains of *B. pumilus* were sequenced, with two then being fully assembled^18^. These ten strains mainly showed industrial or medical merits by producing bioactive substances or potential antiviral agents. However, no genome of strains associated with plant or human disease was sequenced^18^,^19^,^20^,^21^. The genomic information of pathogenic strains of *B. pumilus* will be important for developing an understanding on the pathogenesis and to identify potential control strategies for pathogenic *B. pumilus*. The *B. pumilus* strain GR8 isolated from Shandong Province of China is an opportunistic pathogen of ginger and can cause severe rot symptoms of ginger rhizomes^22^. In this study, we characterized the genome of this strain. Analysis of the GR8 genome sequence provided insight into both the pathogenesis of *B. pumilus* and possible novel control strategies, and made the bio-safety aspects of *B. pumilus* as plant growth-promoting rhizobacteria (PGPR) debatable.

## Results

### Genome sequence and general features of the *B. pumilus* strain GR8

**Figure 1:**
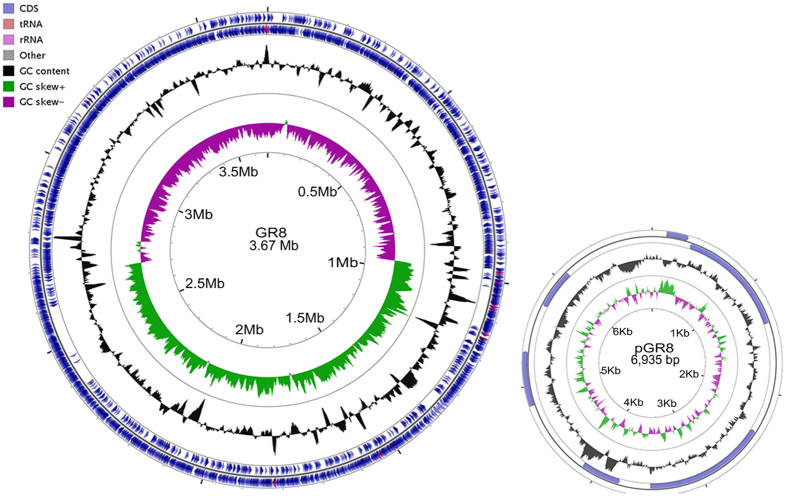
Circular genome maps of the *B. pumilus* strain GR8 chromosome and the plasmid pGR8. The genome of GR8 was annotated using the NCBI Prokaryotic Genome Annotation Pipeline (PGAP) and visualized using the CGView Server.

The genome of GR8 was sequenced using the Illumina HiSeq 2500 and was *de novo* assembled into 10 contigs with an average sequencing depth of approximately 120-fold. The gaps between contigs were filled by primer walking. The complete circular genome of GR8 was 3,674,489 bp in length with a G+C content of 41.42% (Fig. 1), and encoded 3,713 ORFs. This genome was the third completely sequenced *B. pumilus* genome. A comparison of general genome features of ten sequenced strains of *B. pumilus* and the strain GR8 is summaried in Table 1. Compared with the other sequenced *B. pumilus* genomes, GR8 had a smaller genome size and possessed more tRNA and rRNA encoding genes (81 and 24, respectively). One plasmid sequence was also obtained (Fig. 1; Supplementary Fig. S1). The length of the plasmid, named pGR8, was 6,935 bp with a G+C content of 36.82%, and it encoded six ORFs. The sequence of pGR8 exhibited 53% and 61% similarity to the *B. subtilis* plasmid pPOD2000 and the *B. pumilus* plasmid pPL7065, respectively.

### Comparative genomic analysis

**Figure 2:**
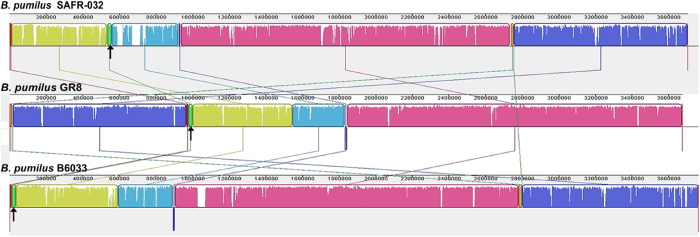
Comparative genome analysis of three completely sequenced strains of *B. pumilus*. The genome sequences of SAFR-032, GR8 and B6033 were compared using progressive Mauve. The rearrangement regions in the genomes are indicated with black arrows.

The eleven strains of *B. pumilus* from which genomes have been sequenced had a wide biogeographical distribution, while they exhibited highly similar genome features (Table 1). Comparative genomic analysis showed that the genome of GR8 exhibited high similarity to the genome of strain B6033 isolated from India (Fig. 2; Supplementary Fig. S2). Sequence comparisons among the three completely sequenced genomes of the *B. pumilus* (strains GR8, B6033 and SAFR-032), showed widespread co-linearity, except for a 24-kbp genome rearrangement from 976,679 nt to 1,000,676 nt in GR8 genome. The rearrangement region in GR8 genome was predicted to encode proteins with functions of genome replication and nucleotide metabolism. The encoding genes of two recombinases, RecF and RecR, were found at the 5’-terminus and the 3’-terminus of the rearrangement region, respectively, and might be essential for the recombination of this region.

### Plant cell wall-degrading enzymes

Plant pathogens infect plants by employing plant cell wall-degrading enzymes (PCWDEs) to destroy plant cells^23^. Numerous putative proteins associated with the degradation of the plant cell wall were predicted to be encoded by the genome of the ginger rhizome rot disease pathogen GR8. The PCWDEs were mainly CAZymes, wiht 452 CAZyme genes identified in the GR8 genome. The predicted CAZymes included five categories as follows: carbohydrate esterase (CE), glycosyltransferase (GT), glucoside hydrolases (GH), polysaccharide lyase (PL) and carbohydrate-binding molecules (CBMs) (Supplementary Fig. S3). In the CE class, the abundance of the acetyl xylan esterases, CE1 and CE4 (representing 22% and 15%, respectively), was high, but the abundance of CE1 and CE4 was less than that of CE11 with an abundance of 29%. Cellulose (GT2) was the most abundant (representing 54%) in the GT class, and chitinase (GH18) represented the most abundant glucoside hydrolase (GH) followed by galactanase (GH53), mannosidase (GH92 and GH1), and glucosidase (GH1), which all degrade plant cell wall components^23^. Polysaccharide lyase (PL) represented the CAZymes that degraded pectin, the major component of plant cell walls^24^. Three types of the predicted PLs (PL1, PL9 and PL10) were all pectate lyases. Carbohydrate-binding molecules (CBMs) are responsible for the attaching of enzymes to cellulose, the major component of plant cell walls. CBM2 and CBM3 were the second and third most abundant CBMs identified. Overall, there were fewer CAZyme genes in the GR8 genome compared to the genomes of strains B6033 and SAFR-032 (Supplementary Fig. S4).

### Plant pathogenicity gene candidates

**Figure 3:**
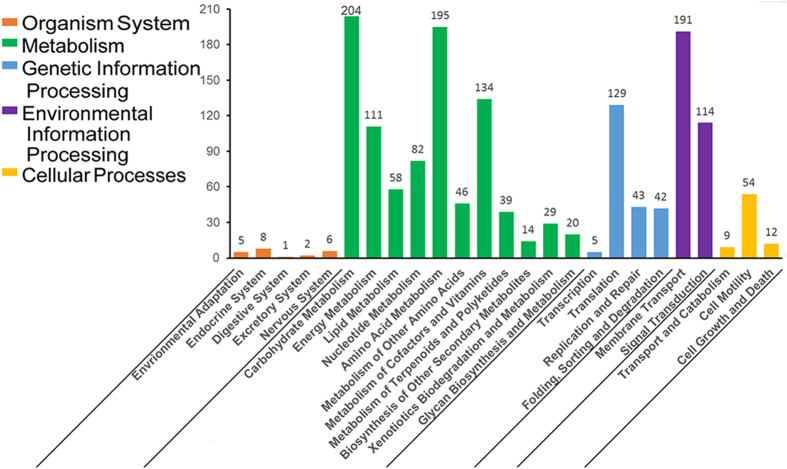
Functional classification of proteins encoded by the GR8 genome. The functions of the proteins were assigned using KAAS. The number of each class is indicated above the corresponding column.

The putative functional classifications of the proteins encoded by GR8 genome were analyzed using KAAS^25^. In total, 1,553 putative proteins (representing 43.2% of ORFs) were associated with the processes of metabolism, environmental information processing and genetic information processing (Fig. 3).

Four putative proteins from the GR8 genome, including three flagellin FliC proteins and one elongation factor Tu (EF-Tu), were classified as environmental adaptation proteins. These proteins exhibit functions in the interaction between bacterial pathogens and plants. They can also induce the immunity of the plant host to bacterial pathogens, and the flagellin also facilitates the adherence of plant pathogens to plants by absorbing plant lignin^26^,^27^.

Two putative proteases, with similarity to the human pathogen protease responsible for degradation of protein in the mucus layer during infection^28^, were found to be encoded by the GR8 genome. The GroEL and LepA encoding genes were also found in the GR8 genome. GroEL is a surface-located chaperonin which plays a role in pathogen entry or the entry of the bacteria-containing vacuole to the host cell^29^,^30^. The LepA protein aids the exit of bacteria from the host cell to allow infection of neighboring cells^29^. Similar proteins encoded by the GR8 genome might facilitate the invasion of the strain into ginger rhizome parenchyma cells.

Numerous proteins, which may be associated with the adaptation of the strain to the ginger parenchyma tissue extracellular space environment, were predicted from the GR8 genome as follows: NsrR, Hmp, RocF, DltA, DltB, DltC, DltD, MprF, BceA, BceB, PstS, DnaK, LuxS and SdhA. NsrR, Hmp and RocF might inhibit the production of NO or transform NO into nitro. The accumulation of NO in plant cells acts to induce the plant defense system against plant pathogens, and NO is also a toxicant to pathogenic bacteria^31^,^32^. The production of antimicrobial peptides is a crucial way for plant hosts to prevent infection and colonization by bacterial plant pathogens. A three-component system that provides Gram-positive strains with resistance to plant-produced antimicrobial peptides has been well characterized^33^. The system is composed of a *dl*t system (DltA/DltB/DltC/DltD), an MprF protein and an ABC transporter (BceA/BceB). The related genes of the three-component system were found in the genome of GR8 (Table 2). The GR8 genome also encoded PstS and DnaK proteins, which may help GR8 to adapt to the low phosphorus environment of the extracellular space of ginger parenchymatous tissue^34^. The formation of biofilm is a strategy bacterium utilize to enhance infective capability, and LuxS has been reported to mediate biofilm formation^35^. Biofilm formed by GR8 was observed during daily culture of the strain. According to previous reports, GR8 could invade parenchymatous cells and live within the host by using the nutrition of the cells^22^. Two proteins, PstS and SdhA, which inhibit host cell apoptosis^30^, were encoded by the GR8 genome and these might benefit the intracellular life cycle of GR8.

### Phage-resistance system and prophage regions of GR8

Because phage and phage endolysin can lyse bacteria, they are thought of as potential antimicrobial agents against plant bacterial pathogen^36^,^37^. To avoid phage infection, bacteria have also evolved defense systems, such as resistance to phage absorption, penetration blocks, restriction modification (R-M) system and clustered regularly interspaced short palindromic repeats (CRISPR/Cas) system^38^. The CRISPR/Cas system is a general adaptive immunity system that protects the bacterium from foreign phage DNA and widely exists in the prokaryotic organism^39^,^40^. The GR8 genome was analyzed by the CRISPR finder online service, and no CRISPR/Cas system was found. The R-M system in the GR8 genome was also analyzed by comparing the genome sequence with the REBASE database, and the result showed that only one type I R-M system was found. The R-M system recognized the specific sites on the foreign DNA and cleaved the DNA without modification^41^. According to previous reports, strain GR8 could be lyzed by kinds of phages^42^. The reason might be the lack of phage defense system in the GR8 genome. This result suggested that phage might be a promising antimicrobial agent for the control of ginger rhizome rot disease caused by strain GR8.

**Figure 4:**
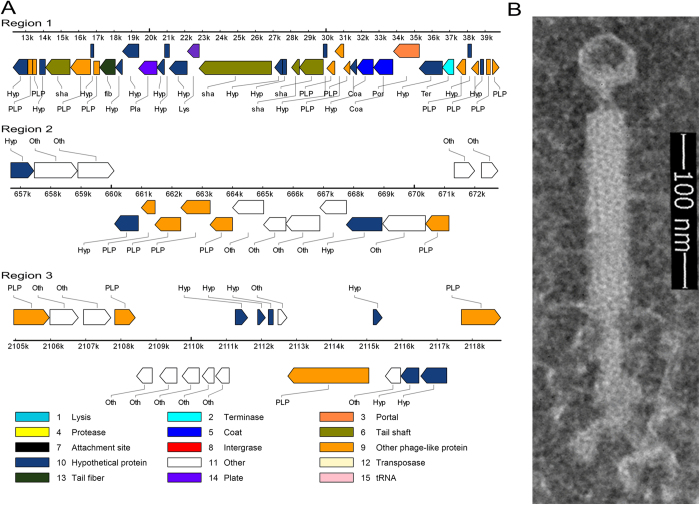
Analysis of prophages in the GR8 genome. The prophage genome in the GR8 chromosome was predicted using PHAST. (**A**) Schematic diagrams of the three predicted prophage regions (region 1, region 2, and region 3) in the GR8 genome. The location of each prophage region in the GR8 genome and the functional classification of the predicted prophage proteins are indicated. (**B**) Morphology of phage particles induced from GR8. The phage particle was negatively stained with 2% potassium phosphotungstate and observed by transmission electron microscopy.

The prophage region in the GR8 genome was evaluated using PHAST and three putative prophages, including an intact prophage (region 1), an incomplete prophage (region 2) and a questionable prophage (region 3), were found (Fig. 4A). The intact prophage region was located between sites 12,281 to 39,856 nt with a G+C content of 41.47%, which was similar to the G+C content of the GR8 genome, and it exhibited 87% genomic similarity to the genome of the defective phage, PBP180, from *B. pumilus* strain CCTCC AB94180^43^. The GR8 strain was inducible by mitomycin C, but the induced phage could not infect the GR8 strain (Supplementary Fig. S5). The induced supernatants were observed by transmission electron microscopy (TEM). Phage particles had an isometric head with a diameter of 27 nm, a neck (19 nm in length), a contractile tail (189 nm in length and 16 mm in width), and a tail needle (60 nm in length) (Fig. 4B). The phage particles were purified, and the DNA was extracted as described^44^. Restriction enzyme digestions of the DNA extracted from the phage particles showed that the DNA could not be digested into separated bands (Supplementary Fig. S6). According to previous reports, the prophage PBSX induced from *Bacillus subtilis* could randomly pack chromosomal DNA^45^. We speculated that the induced phage from GR8 exhibited the same feature as prophage PBSX and the capsids packed the chromosomal DNA randomly.

## Discussion

As a widely used bacterium for producing bioactive substances with industrial merits or used as a plant growth-promoting rhizobacterium, the biosafety of *B. pumilus* has been scarcely evaluated^11^. Although several strains of *B. pumilus* have been identified as pathogens of plants and humans, the pathogenesis and the genetic foundation of pathogenic *B. pumilus* was unknown^11^,^15^,^16^. However, the genetic analysis of pathogenic strains is helpful for understanding pathogenesis and to identify novel control strategies.

**Figure 5:**
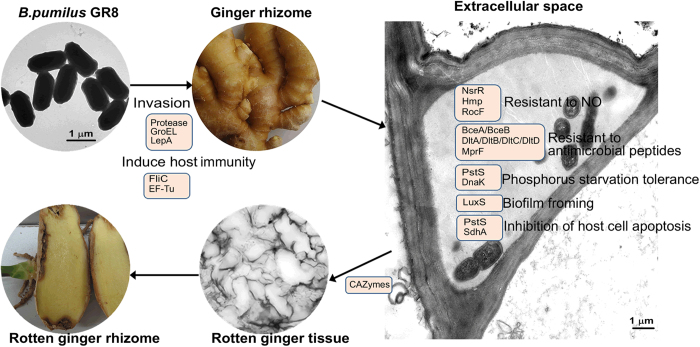
Pathogenicity of *B. pumilus* strain GR8 to ginger rhizome. The putative proteins involved in the infection of ginger rhizome by GR8 are indicated at each stage, and the functions are also shown.

According to previous report, the GR8 was identified as an opportunistic pathogen of ginger, gaining access via wounds and subsequently invading the cells, and causing the rotting of the rhizome^22^. The genomic analysis of GR8 provided an understanding to the pathogenesis of the strain. Several genes were found to be involved in the invasion of GR8 to the ginger rhizome (Fig. 5; Supplementary Fig. S7). After invasion, the GR8 strain was located in the extracellular space of the parenchymatous tissue, and numerous genes were responsible for the adaptation of GR8 in the extracellular space. During the growth of GR8 in the extracellular space, the expression and the secretion of enzymes belonging to the CAZyme families caused the degradation of ginger rhizome cells and finally the breakdown of the ginger rhizome tissue around the wounded site. The gene candidates associated with the pathogenesis of GR8 also existed in the genomes of the other completely sequenced *B. pumilus* strains, B6033 and SAFR-032 (Table 2) and numerous genes among them were associated with human infective diseases caused by bacteria^28^,^30^,^35^. Comparative genomic analysis revealed that the genomes among GR8, B6033 and SAFR-032 were highly similar. The *B. pumilus* strain B6033 was selected for bio-catalyzing the stereospecific oxidation of β-lactams^18^. The SAFR-032 *B. pumilus* strain was isolated from the Spacecraft Assembly Facility at the NASA Jet Propulsion Laboratory, and spores produced by this strain exhibited unusual resistance to UV radiation^46^. The high genome similarity and the wide existence of the putative pathogenic associated genes in the other strains of *B. pumilus* used for industrial purposes make the use of *B. pumilus* debatable, and highlights the need to determine the biosafety of the strains before further industrial use, especially before the use of such strains as plant growth-promoting rhizobacteria.

A three-component system, which might provide resistance to antimicrobial peptides, was found based on the genomic analysis of strain GR8, so the microbe-produced antimicrobial peptides might be useless for controlling ginger rhizome rot disease caused by GR8. Thus, development of effective strategies for controlling the disease caused by strain GR8 is necessary. In the past few years, phage was thought to be a promising novel antimicrobial agent against plant pathogenic bacteria, but the defense systems of the bacteria prevent the infection of phage and cause failure of phage therapy^47^,^48^. The CRISPR/Cas system and R-M system are two general defense systems of the prokaryotic organism against phage infection. Only a type I R-M system was detected in the GR8 genome, and no CRISPR/Cas system was identified. According to a previous report, phage therapy can efficiently control the ginger rhizome rot caused by GR8^42^. The phage defense systems were also absent in the other genome-sequenced strains of *B. pumilus*, thus suggesting that phage therapy might also be also useful in the control of human and plant diseases caused by *B. pumilu*s.

## Methods

### Bacterial strain and culture conditions

The ginger rhizome rot disease pathogen *B. pumilus* strain GR8 was isolated from soil collected around rotten ginger rhizome located in Weifang City of Shandong Province in China and stored by our laboratory. The strain was grown in Luria-Bertani (LB) broth at 30 °C for 12 h with moderate shaking. The cells were then harvested for genomic DNA extraction. The pathogenesis of *B. pumilus* strain GR8 to the ginger rhizome was analyzed as previously described^22^.

### Genome sequencing and annotation

The genome of GR8 was extracted as previously described^49^, and randomly sheared and purified to construct a paired-end library with an insert size of 300 bp. The constructed genome library was then sequenced using Illumina HiSeq2500 (Illumina, California, USA). The genome was assembled into contigs using Velvet v1.2.07^50^ software, and the gaps between contigs were filled by primer walking. The complete genome of GR8 was annotated using the Prokaryotic Genomes Annotation Pipeline (PGAP; http://www.ncbi.nlm.nih.gov/genome/annotation_prok/) at NCBI.

### Bioinformatic analysis

The functions of the proteins were classified by searching the amino acid sequences of the putative proteins against the KEGG Automatic Annotation Server (KAAS, http://www.genome.jp/tools/kaas/). Comparative genomic analysis of the genomes was performed using progressive Mauve^51^. The CRISPR/Cas system in the genome was identified using the CRISPR Finder server^52^, and the R-M system in the genome was analyzed based on the data of REBASE^41^. Carbohydrate-active enzymes (CAZymes) were predicted with the CAZymes Analysis Toolkit (CAT v1.8)^53^. The genome was visualized using CGView^54^.

### Prophage induction

Mitomycin C (ranging from 0.1 to 1.0 μg/ml with intervals of 0.1 μg/ml) was added to the culture of GR8 at the exponential growth stage in LB broth with a turbidity of 0.8 at 600 nm. After growing for 8 hours, the turbidity at 600 nm of the cultures was detected, and the culture supernatants were collected. The infective ability of the induced phages in the supernatants was determined using the double agar overlay assay method as described before^44^. The induced phage particles were negative stained with 2% potassium phosphotungstate and were observed by transmission electron microscopy (TEM) (H-7000FA, HITACHI, Tokyo, Japan).

### Accession numbers

The chromosome sequence of *B. pumilus* GR8 and the plasmid sequence of the *B. pumilus* GR8 plasmid (pGR8) were deposited in the NCBI database under the accession numbers CP009108 and CP009109, respectively.

## Author Contributions

M.G. conceived and designed the project; Y.H. performed the experiments and analyzed the data; Y.H. drafted the manuscript; M.G. reviewed, revised, and edited the manuscript. All authors have read and approved the final manuscript.

## Additional Information

**How to cite this article**: Yuan, Y. and Gao, M. Genomic analysis of a ginger pathogen *Bacillus pumilus* providing the understanding to the pathogenesis and the novel control strategy. *Sci. Rep.* **5**, 10259; doi: 10.1038/srep10259 (2015).

## Supplementary Material

Supplementary Information

## Figures and Tables

**Table 1:** Comparison of general genome features of sequenced strains of *B. pumilus*.

**Feature**	***Bacillus pumilus*** **strains**										
	**GR8**	**B6033**^18^	**SAFR-032**^47^	**S-1**^2^	**7p**^20^	**CCMA-560**^19^	**Fairview**^55^	**3-19**^a,^^56^	**ATCC7061**^47^	**INR7**^56^	
Origin of strain	China	Inda	USA	China	Russia	Brazil	Australia	Russia	USA	Korea	China
Chromosome size (bp)	3,674,489	3,763,493	3,704,465	3,692,073	3,573,949	3,848,811	3,838,013	3,573,949	3,833,998	3,681,709	3,747,698
G+C content (%)	41.4	41.4	41.3	41.3		41.8	41.5	41.9	41.7	41.3	41.3
Number of genes	3,713	3,807	3,822	3,708	3,596	3,594	3,784	3,563	4,162	3,725	6,756
No. of CDS	3,592	3,659	3,679	3,643	3,460	3,460	3,717	3,363	3,900	3,625	3,676
No. of pseudogenes	15	42	50	11		57	41	123	190	30	12
No. of rRNA	24	24	21	4	4	4	3	4	7	4	21
No. of tRNA	81	81	69	49	72	72	22	72	58	65	46
No. of other RNA	1	1	3	1		1	1	1	7	1	1
GenBank accession	CP009108	CP007436	CP000813	AGBY00000000	JHUD00000000	AUYP01000000	JFBY01000000	JOJX00000000	ABRX00000000	AYTK00000000	AMDH00000000

^a^3-19 is derived from *B. pumilus* strain 7P.

**Table 2:** Plant pathogenic gene candidates in the *B. pumilus* strain GR8 genome and corresponding genes in the genomes of strains B6033 and SAFR-032.

**Function during GR8 infection**	**Protein**	**Gene for GR8**	**Corresponding gene for B6033**	**Corresponding gene for SAFR-032**	**Description**
**Induce host immunity**	FliC	ID12_11065	BW16_06360	BPUM_0150	Induce host immunity and aid adherence^27^
	FliC	ID12_11180	BW16_06550	BPUM_1149	
	FliC	ID12_11185	BW16_06555	BPUM_1152	
	EF-Tu	ID12_05735	BW16_00715	BPUM_0099	
**Invasion**	Protease	ID12_17480	BW16_12860	BPUM_2370	Degrade protein^28^
	Protease	ID12_17485	BW16_12865	BPUM_2371	
	GroEL	ID12_08120	BW16_03100	BPUM_0535	
	LepA	ID12_17000	BW16_12350	BPUM_2284	
**Resistance to NO**	NsrR	ID12_09830	BW16_04850	BPUM_0893	NO detoxification^32^
	Hmp	ID12_09835	BW16_04855	BPUM_0894	
	RocF	ID12_06065	BW16_01045	BPUM_0159	
**Resistance to antimicrobial peptides**	BceA	ID12_18605	BW16_13990	BPUM_2594	Antimicrobial peptide transport^33^
	BceB	ID12_18610	BW16_13995	BPUM_2595	
	DltA	ID12_18030	BW16_13390	BPUM_2479	
	DltB	ID12_18025	BW16_13395	BPUM_2478	
	DltC	ID12_18020	BW16_13400	BPUM_2477	
	DltD	ID12_18015	BW16_13405	BPUM_2476	
	MprF	ID12_09195	BW16_04235	BPUM_0787	
**Phosphorus starvation tolerance**	PstS	ID12_16725	BW16_12070	BPUM_2227	Phosphate binding^34^
	DnaK	ID12_16980	BW16_12330	BPUM_2280	
**Biofilm forming**	LuxS	ID12_19195	BW16_14590	BPUM_2705	Mediate biofilm forming^35^
**Inhibition of host cell apoptosis**	PstS	ID12_16725	BW16_12070	BPUM_2227	Inhibit host cell apoptosis^30^
	SdhA	ID12_18145	BW16_13530	BPUM_2502	
