# Supplementary material for: Genomic analysis of a ginger pathogen Bacillus pumilus providing the understanding to the pathogenesis and the novel control strategy
Source: Sci Rep. 2015 May 19;5:10259. doi: 10.1038/srep10259 (PMC4437294; doi:10.1038/srep10259)
Supplement: Supplementary Information [file srep10259-s1.pdf]

Genomic analysis of a ginger pathogen *Bacillus pumilus* providing the understanding to the pathogenesis and the novel control strategy

Yihui Yuan, Meiying Gao*

Key Laboratory of Agricultural and Environmental Microbiology, Wuhan Institute of Virology, Chinese Academy of Sciences, Wuhan, P.R. China

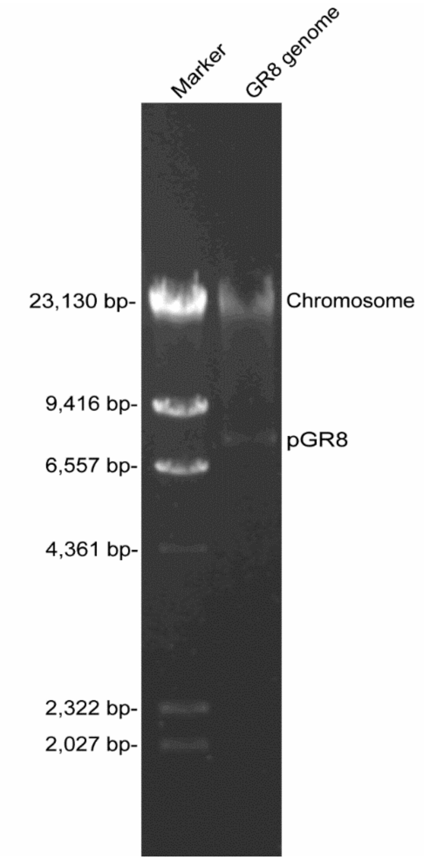

Figure S1 Genome analysis of GR8 genome. The chromosome and the plasmid pGR8 in GR8 genome were indicated.

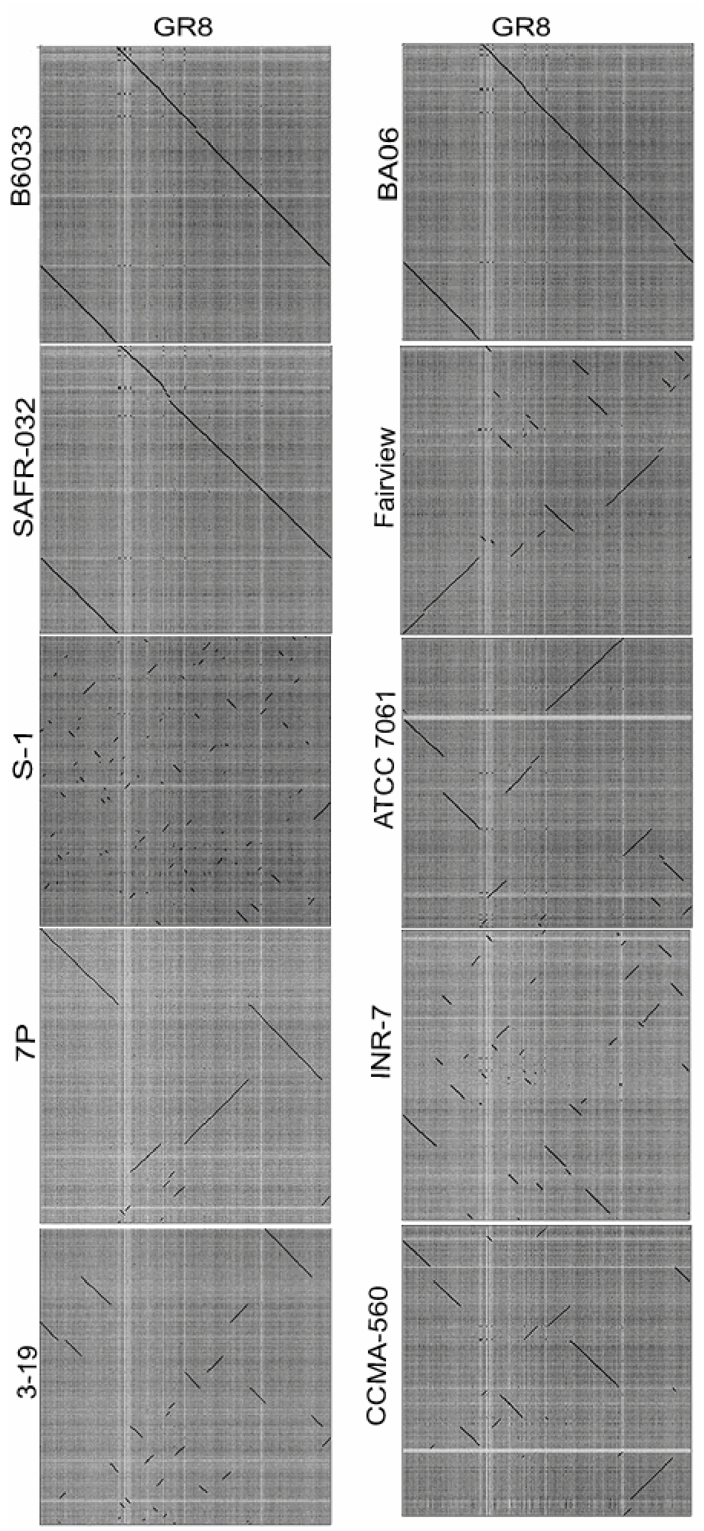

Figure S2 Comparative genome analysis of *Bacillus pumilus* GR8 and other sequenced *B. pumilus* strains. The dot plot analysis of the genomes was carried out by using Gepard (Version 1.30, http://www.helmholtz-muenchen.de/icb/gepard) and the strain names were indicated.

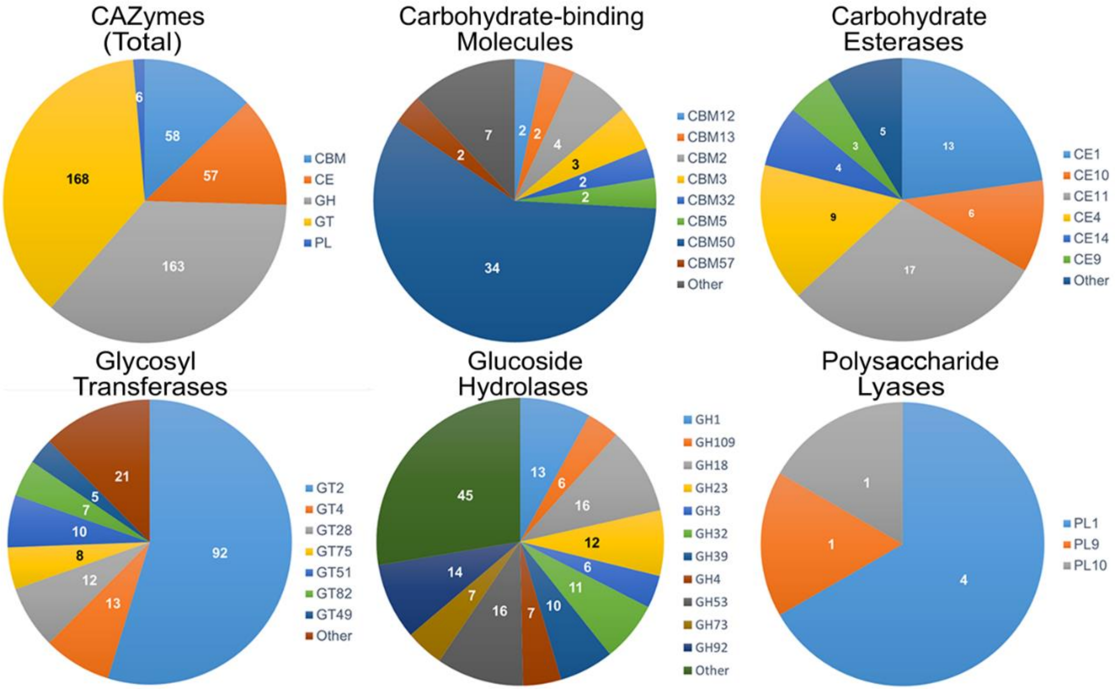

Figure S3 CAZymes predicted by *B. pumilus* GR8 genome . The five categories of CAZymes, including carbohydrate-bingding molecules (CBMs), carbohydrate esterases (CEs), glycosyl transferases (GTs), glucoside hydrolases (GHs) and polysaccharide lyases (PLs) were also presented.

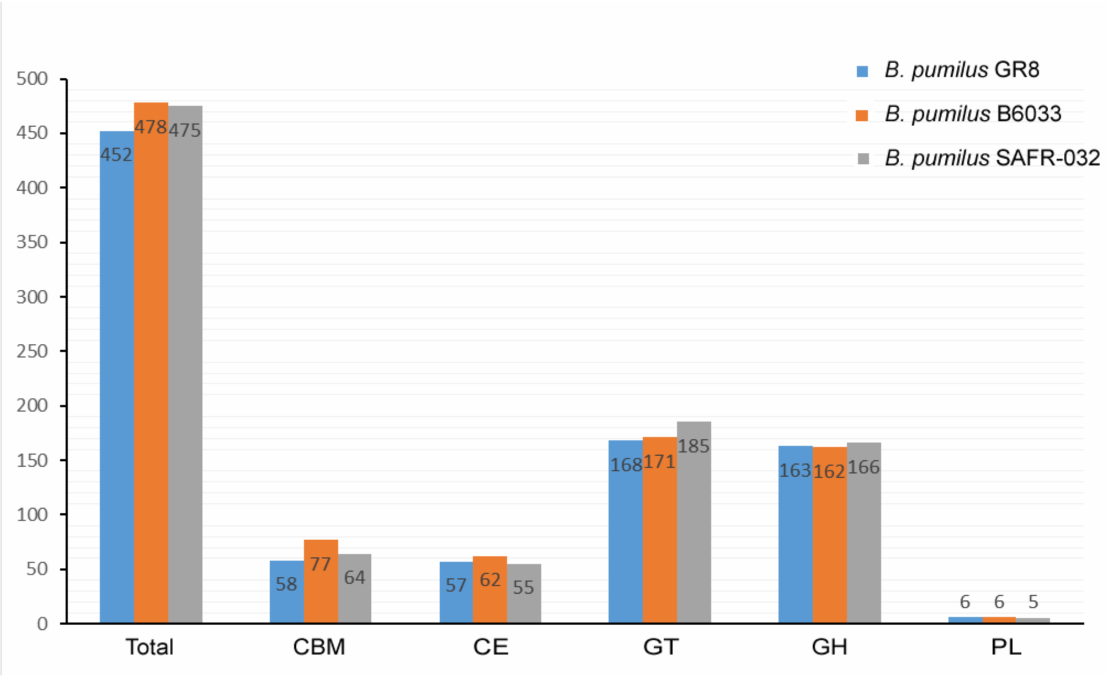

Figure S4 Summary of proteins encoded by *B. pumilus* strain GR8, B6033, and SAFR-032 genomes assigned with CAZyme functional annotations. The total numbers of the proteins annotated as CAZymes was indicated and the individual summary of each category was also shown. The five CAZyme categories were carbohydrate-bingding molecules (CBMs), carbohydrate esterases (CEs), glycosyl transferases (GTs), glucoside hydrolases (GHs) and polysaccharide lyases (PLs).

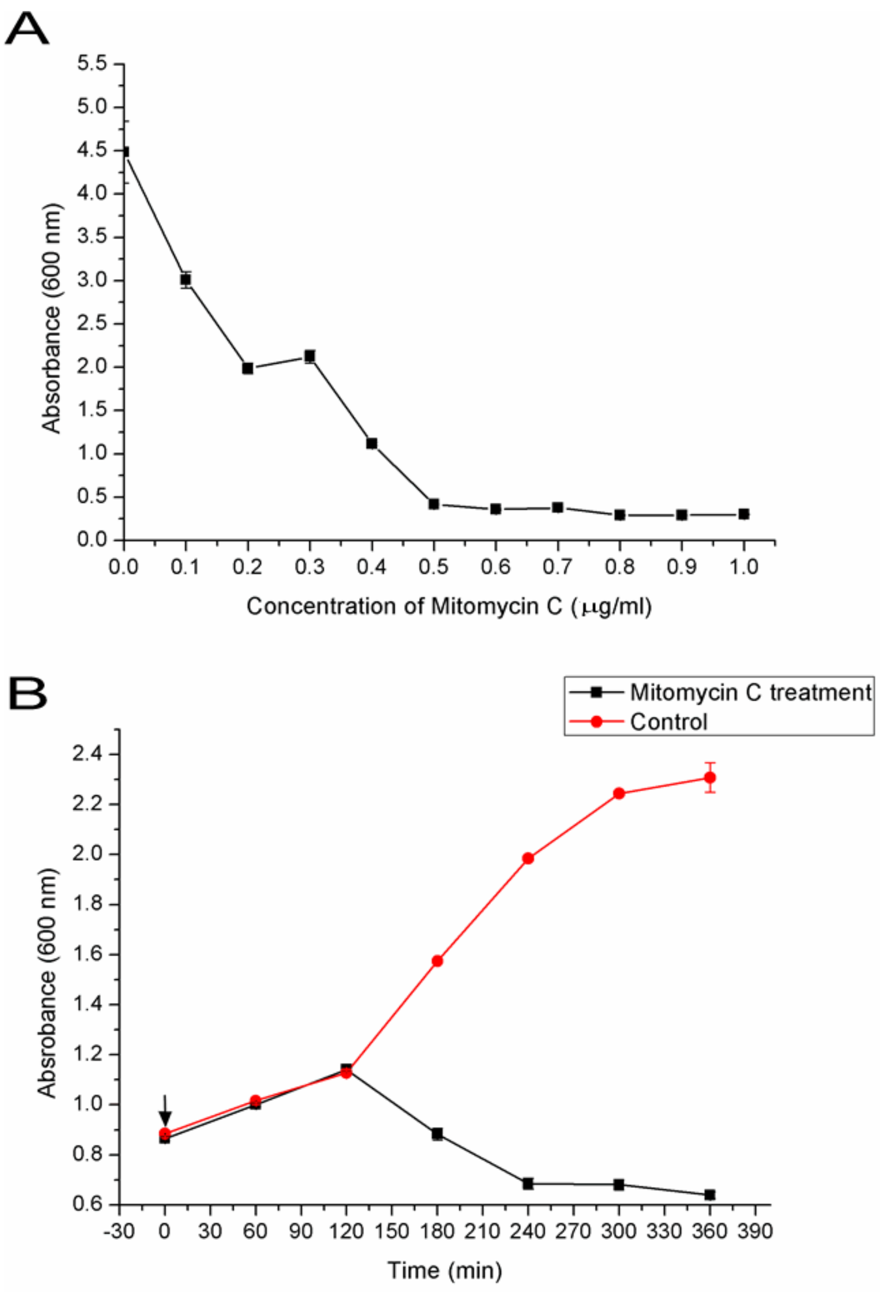

Figure S5 Induction of the prophage from *B. pumilus* GR by mitomycin C. (A) GR8 was induced with different concentration of mitomycin C. (B) Growth curve of GR8 induced by mitomycin C. The black arrow indicated the time point of mitomycin C added and the mitomycin C was added into the cultures to a final concentration of 0.5 μg/ml. The sterile water was used as control. The experiment was carried out in triplicate.

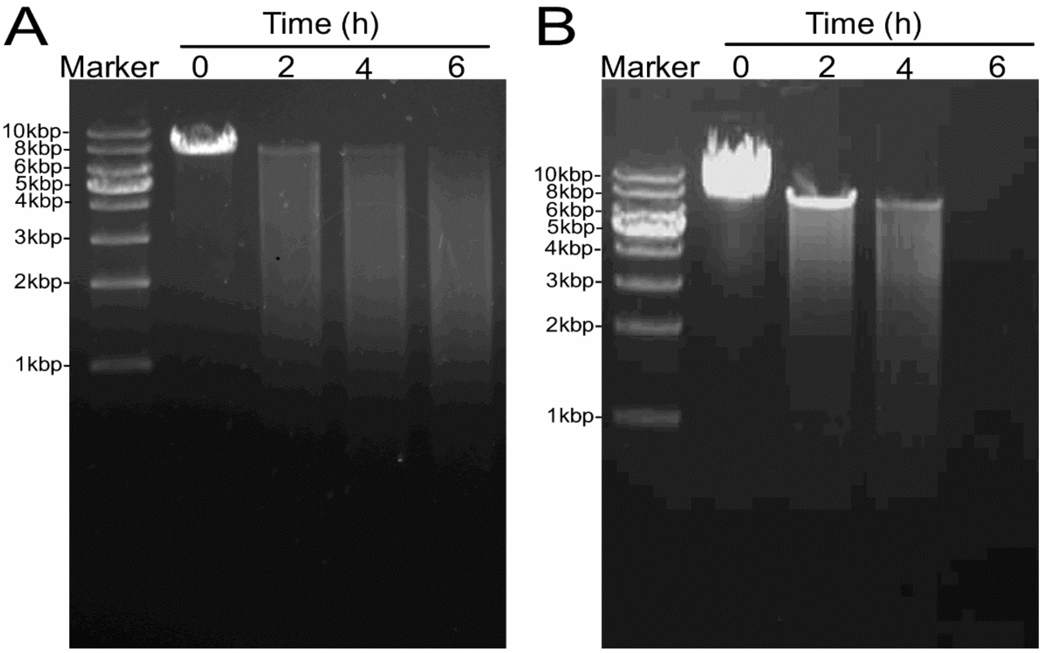

Figure S6 Restriction enzyme digestion of the DNA from the induced phage of GR8. The DNA was digested by *EcoR*I (A) and *BamH*I (B), respectively. The samples were collected every two hours and analyzed by 0.8% agarose gel electrophoresis.

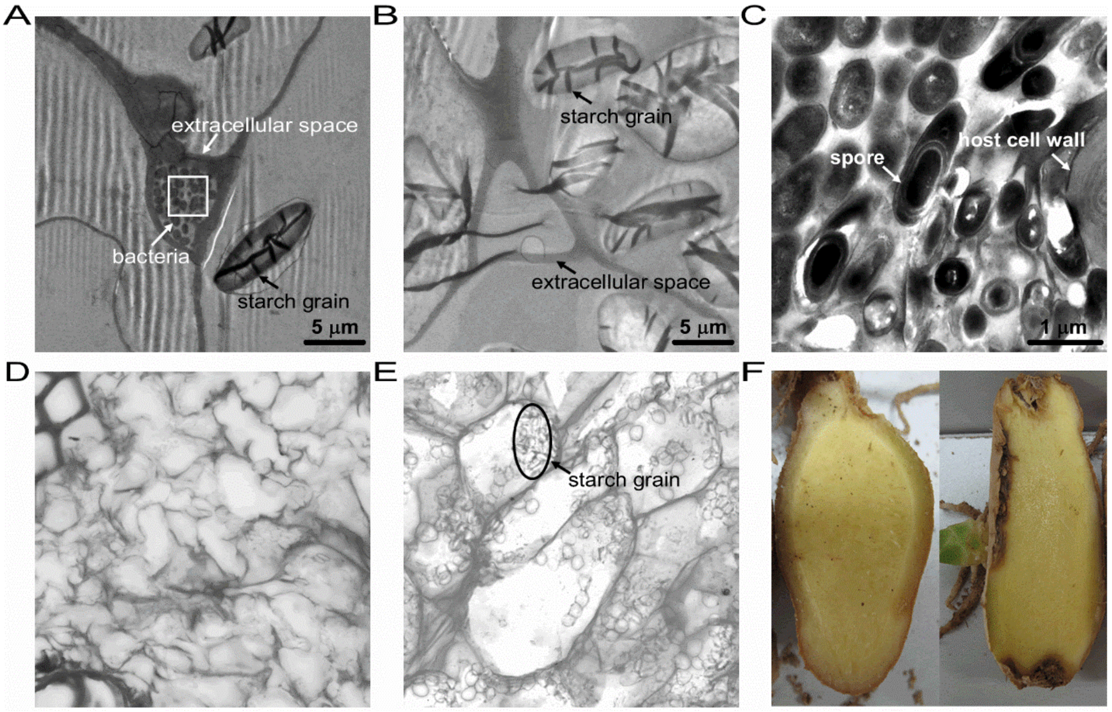

Figure S7 Histological observation of the ginger rhizome. (A) and (B), The rotten and the health ginger rhizome sections were observed by TEM, respectively. The bacteria growth in the extracellular space were indicated. (C), The strain GR8 cells and spores formed in the extracellular space of the parenchymatous tissue was observed by TEM. (D) and (E), The rotten and health the ginger rhizome sections was observed by LM, respectively. (F), The left panel and the right panel indicated the health and the rotten ginger rhizome, respectively. The histological change of the rotten ginger rhizome was observed by TEM (H-7000FA, HITACHI, Tokyo, Japan) and LM (CX41, Olympus, Tokyo, Japan).
